# Impact of examined lymph node number on accurate nodal staging and long-term survival of resected Siewert type II-III adenocarcinoma of the esophagogastric junction: A large population-based study

**DOI:** 10.3389/fonc.2022.979338

**Published:** 2022-10-31

**Authors:** Baicheng Ding, Jiahui Yong, Lixiang Zhang, Panquan Luo, Endong Song, Abigail N. Rankine, Zhijian Wei, Xingyu Wang, Aman Xu

**Affiliations:** ^1^ Department of Emergency Surgery, The First Affiliated Hospital of Anhui Medical University, Hefei, China; ^2^ Department of Transfusion, The First Affiliated Hospital of University of Science and Technology of China (USTC), Division of Life Sciences and Medicine, University of Science and Technology of China, Hefei, China; ^3^ Department of Gastrointestinal Surgery, Department of General Surgery, The First Affiliated Hospital of Anhui Medical University, Hefei, China; ^4^ Department of Clinical Medicine, Anhui Medical University, Hefei, China

**Keywords:** adenocarcinoma of the esophagogastric junction (AEG), Siewert type, examined lymph node number, accurate nodal staging, long-term survival, optimal cut point

## Abstract

**Background:**

We aimed to investigate the association between the number of examined lymph nodes (ELNs) and accurate nodal staging and long-term survival in Siewert type II-III Adenocarcinoma of the Esophagogastric Junction (AEG) by using large population-based databases and determined the optimal ELN number threshold.

**Methods:**

Data on Stage I-III Siewert type II-III AEG patients from 2010 to 2014 respectively from the United States (US) SEER database and a Chinese large medical center institutional registry were analyzed for correlation between the ELN number and stage migration (node negative-to-positive) and overall survival (OS) by using multivariable-adjusted logistic and Cox regression models, respectively. The series of odds ratios (ORs), and hazard ratios (HRs) were fitted with a LOWESS smoother, and the structural breakpoints were determined by Chow test. The selected optimal cut point was then validated with the 2015 to 2016 SEER database.

**Results:**

Both the US cohort(n=1387) and China cohort(n=981) showed significantly increases from node-negative to node-positive disease (OR_theUS_1.032,95%CI 1.017–1.046;OR_China_1.034,95%CI 1.002–1.065) and enhancements in overall survival (HR_theUS_0.970,95%CI 0.961-0.979;HR_China_0.960,95%CI 0.940-0.980) with the increasing ELN number after controlling for confounders. Associations for both stage migration and overall survival were still significant in most subgroups’ stratification. Cut point analysis showed a threshold ELN number of 18, which was validated both in the cohorts where it originated and in an independent SEER data cohort(n=379).

**Conclusions:**

More ELNs are associated with accurate nodal staging(negative-to-positive) as well as higher overall survival in resected Siewert types II-III AEG, We recommend 18 ELNs as the optimal cut point for the quality assessment of postoperative lymph node examination or prognostic stratification in clinical practice.

## Background

The incidence of Adenocarcinoma of the Esophagogastric Junction(AEG)has increased rapidly globally over time (1, 2), and the specific anatomical location causes its biological behavior to be different from both esophageal and gastric cancers. Tumor classification based on the anatomical location of the tumor epicenter relative to the esophagogastric junction (EGJ) by Siewert et al. is widely accepted. The diagnosis and treatment of Siewert type I (5-1cm above the EGJ) and type III (2-5cm below the EGJ) have reached a worldwide consensus, while Siewert type II (1cm above to 2cm below the EGJ) has always attracted attention and controversy (3, 4). For resected Siewert type II AEG, resection of the primary tumor and regional lymph nodes remains the cornerstone of standard treatment (5).

Positive lymph node (PLN)is one of the most important factors affecting patient prognosis and treatment decisions. Appropriate lymphadenectomy can not only determine the degree of disease through lymph node involvement and provide the basis for postoperative adjuvant therapy, but also remove potential metastatic lymph nodes to improve the therapeutic effect. For example, for patients with esophagogastric junction tumors who have not received preoperative chemoradiation or chemotherapy. Surveillance is recommended for patients with R0 resection and node-negative disease, and for patients with R0 resection and node-positive disease, chemoradiotherapy or chemotherapy is recommended (3).

Previous studies have shown contradictory results on the association of examined lymph nodes (ELNs) and long-term survival. A study by Peng Jun et al. (6) showed that the number of resected lymph nodes ≥12 was an independent predictor of survival for Siewert type II AEG. Some retrospective studies on AEG demonstrated a high prognostic relevance of the lymph node ratio (LNR) but not of the absolute number of lymph nodes (7, 8). Another study on Western populations showed no significant difference in survival between the extended and the less extended lymphadenectomy (9). Most previous studies may have limitations, such as lack of adjustment for confounders, lack of stratified analysis, and insufficient sample size, resulting in less robust results.

For many cancers, such as lung and pancreatic cancer, the minimum or optimal ELN number was highlighted and determined based on the well-studied association between ELNs and stage migration and survival (10, 11). A recent multicenter database-based study has used robust data to determine the minimum numbers of ELNs for accurate nodal staging and optimal survival of stage T1-2 esophageal squamous cell carcinoma(ESCC) patients were 14 and 18, respectively. This study allows patients with localized early-stage disease (stage T1-2) to obtain the greatest clinical benefit from lymphadenectomy (12). However, the current AEG guidelines do not recommend the optimal number of lymph nodes to be removed or examined yet, and some scholars (3, 4, 13) agree that Siewert type I and Siewert type III AEG should be assigned to esophageal cancer and gastric cancer, respectively. For Siewert type II AEG, current research is mostly focused on the surgical method, the characteristics of lymph node metastasis and the quality control of lymph node dissection (14–17), the optimal number of lymph nodes to examine for accurate staging or to improve patient survival has not been well established.

To address these controversial questions, and to more accurately describe the real-world situation, we performed an analysis of large databases in two different regions to further confirm the relationship between ELNs and stage migration and long-term survival. We used multivariate analysis to determine the optimal threshold for ELN numbers.

## Materials and methods

### Data source

Data on Siewert type II-III AEG patients were obtained from the United States (US) SEER database (https://seer.cancer.gov/) and an institutional registry of a large medical center in China. The SEER database, which covered approximately 30% population of the US, is an open-access database containing demographic information, cancer incidence, treatment descriptions, and survival rates which could be obtained by using the SEER*Stat 8.3.9.2 program. The registry of The First Affiliated Hospital of Anhui Medical University from 2010 to 2014, a large medical center in China, provided the data of cancer patients with the approval of the Ethics Review Committee.

### Data collection and definitions

The patient population from the US and China with microscopically confirmed primary invasive Stage I-III Siewert type II-III AEG who underwent surgical resection between the years 2010 and 2014 was eligible for this research (**Table 1**). The selection criteria included (a) complete resection (R0: no residual disease at surgical margins); (b) pathologic TNM stage I-III; (c) histologically confirmed AEG. An independent cases cohort of the SEER database from 2015 to 2016 was also retrieved for validation. The Siewert type of the US cohort was confirmed by SEER personnel, while the China cohort was performed by surgeons according to the tumor located in the EGJ area. Patients were staged by using the seventh edition of the TNM classification (18), Patients diagnosed before 2010 were not included due to the incompatibility of the TNM staging version, Histological codes conforming to the International Classification of Diseases for Oncology 3rd edition (ICD-O-3) were selected. Patients with secondary tumors or other malignancies were not included in our study, and we required at least one lymph node to be examined in surgical patients. Patients with stage IV disease were not eligible and we excluded patients who survived less than one month to avoid the impact of perioperative events.

**Table 1:** Inclusion and exclusion codes for the US data.

Category	Code	
	Inclusion	Exclusion
Site	EsophagusGEJunction& C16.0	C16.1, C16.2, C16.3, C16.4, C16.5, C16.6, C16.8, C16.9
Morphology	8140-8147,8160-8162,8180-8221,8245,8250-8507,8514-8551,8571-8574,8576,8940-8941	8000,8010,8012,8013,8020, 8021,8032,8041,8045,8046, 8051,8070-8072,8082, 8083, 8244,8510,8512,8560,8890, 8980
Surgery	30,33,40-42,50-52,60-63,80	00,10-14,20-20,31-32,90,99
Tumor size	001-988except888	000,888,989-999
Behavior	3	0,2

Information on the patient (years of diagnosis, gender, age, ethnicity, follow-up time, survival status), tumor (differentiation, tumor size, TNM stage, ELN, PNL, LNR), treatment (surgery, Neoadjuvant chemotherapy/radiotherapy, adjuvant chemotherapy/radiotherapy) were collected. The ELN count was the total number of regional lymph nodes which were intraoperatively removed by surgeons and postoperatively examined by pathologists. Nodal stage migration was defined as the movement of a group of patients with a lower stage (with nodes negative) into that with a higher stage (with nodes positive) due to the increased detection of PLNs. LNR was the PLN divided by the ELN count. Age and tumor size were divided into four groups according to X-tile software (version 3.6.1). All specimens from the Chinese database were analyzed by pathologists at the First Affiliated Hospital of Anhui Medical University. The pathological evaluation was performed according to the guidelines developed by the National Health Commission of the People’s Republic of China (19).

### Statistical analysis

Variables were presented by mean ± standard deviation, median (interquartile range), or number (percent). Based on the hypothesis that detecting more lymph nodes gives a greater chance of identifying PLNs, quantifying the number of ELNs, stage migration was assessed by correlating the ELN number and the proportion of positive versus negative nodal stage by using the binary logistic regression model, which was adjusted for other potential confounders related to ELN numbers and/or nodal stage before and/or during resection (gender, age group, ethnicity, differentiation, resection type, tumor size, T stage, Neoadjuvant chemotherapy/radiotherapy administration). The Cox proportional hazards regression model was used to determine the impact of ELN number on overall survival time, with adjustment for other potential prognostic factors, including gender, age group, ethnicity, differentiation, resection type, tumor size, T stage, positive nodes count, management of adjuvant chemotherapy/radiotherapy administration. Adding the interaction terms one by one for tested interactions between ELN number and other stratification factors.

Using a beta-binomial distribution fitted the distribution of the percentage of positive metastatic lymph nodes among all patients with at least one positive lymph node, resulting in model parameters estimated as α and β. This set of parameters was then used to apply a formula to calculate the estimated probability of false-negative lymph nodes for each ELNs.

Equation: P(FNm)=Beta(α, β+m)/Beta(α, β) where m=examined lymph node(≥1)

The curves of the probability of PLNs and undetected PLNs as well as the curves of odds ratios (negative-to-positive node stage migration) and hazard ratios (overall survival) of each ELNs compared with one ELN were fitted by using a LOWESS smoother with a bandwidth of 2/3. Structural breakpoints were then determined by Chow test(F test). The breakpoints were considered as the optimal threshold.

All data were analyzed using SPSS 23.0 for Windows and R (version 4.1.0). A two-sided *P* < 0.05 was considered statistically significant.

## Results

### Patient characteristics

**Figure 1:**
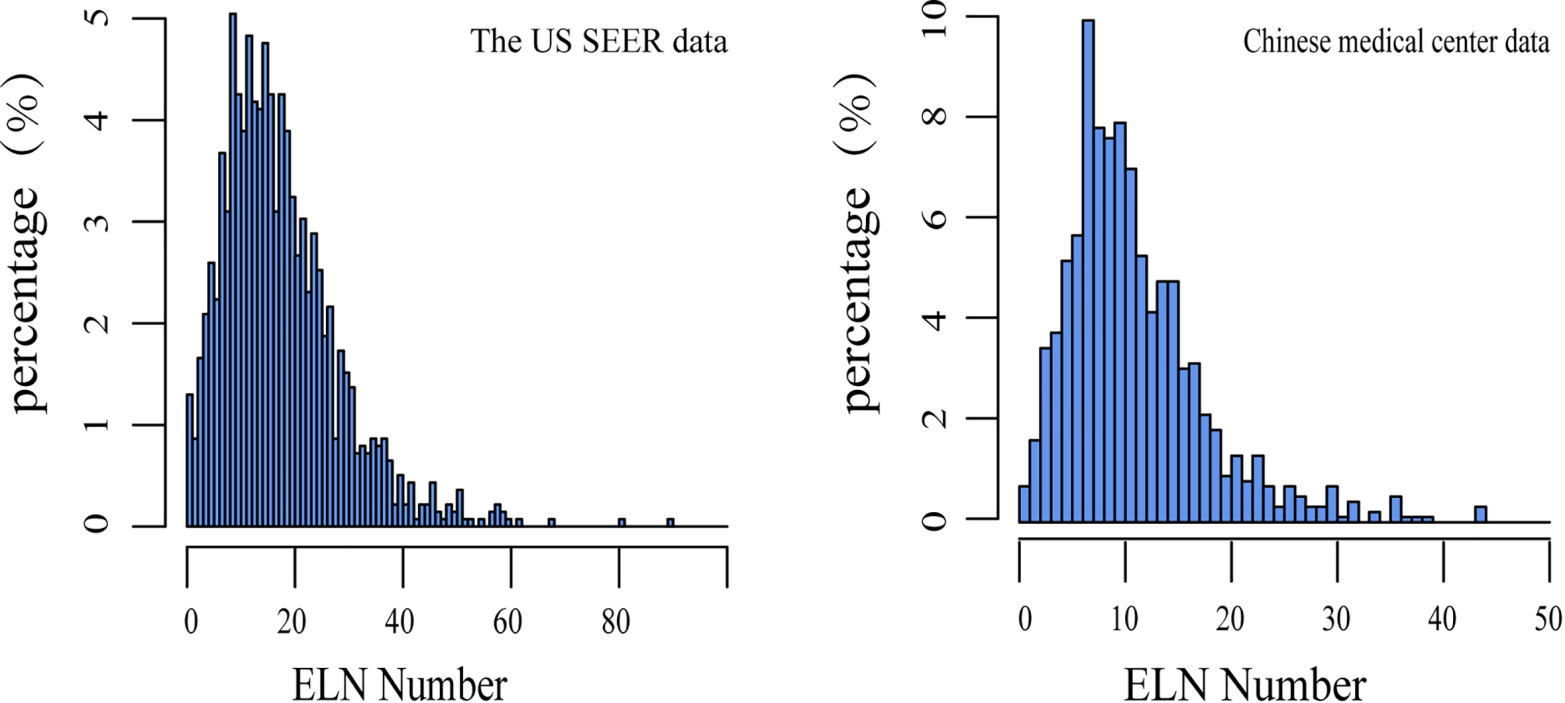
Distribution of the number of ELNs in the US and Chinese data. ELN, Examined lymph node.

The baseline demographics and clinical characteristics analysis of the 1387 patients in the US SEER data cohort and 981 patients in the Chinese medical center data cohort who were eligible for inclusion were presented in **Table 2**. The median follow-up time in the US cohort was 61 months, respectively, compared with 60 months in the China cohort. The mean number of ELNs in the US cohort (18) was more than the China cohort (11), The median PLN number was one in both cohorts. The distribution of ELN numbers in the two cohorts was expressed in **Figure 1**.

**Table 2:** Baseline clinicopathological characteristics.

Characteristic	The US SEER data (2010–2014), n (%)	Chinese medical center data (2010-2014), n (%)
	n=1387	n=981
Gender		
Male	1118 (80.6)	774 (78.9)
Female	269 (19.4)	207 (21.1)
Age (years)		
As continuous	63 ± 11	62 ± 9
<55	303 (21.8)	161 (16.4)
55-65	436 (31.4)	388 (39.6)
65-75	447 (32.2)	343 (35.0)
≥75	201 (14.5)	89 (9.1)
Ethnicity		
White	1193 (86.0)	NA
Black	66 (4.8)	NA
Others#	128 (9.2)	981 (100)
Differentiation		
Well	92 (6.6)	2 (0.2)
Moderately	513 (37.0)	323 (32.9)
Poorly/Undifferentiated	728 (56.4)	656 (66.9)
Surgery		
Partial/subtotal/hemi- gastrectomy	697 (50.3)	50 (5.1)
Near-total/total gastrectomy	305 (22.0)	931 (94.9)
Gastrectomy (NOS)	385 (27.8)	NA
Tumor size (mm)		
As continuous	37 (22,55)	50 (35,70)
<40	718 (51.8)	262 (26.7)
40-60	364 (26.2)	329 (33.5)
>60	305 (22.0)	390 (39.8)
T stage		
T1	267 (19.3)	71 (7.2)
T2	204 (14.7)	75 (7.6)
T3	831 (59.9)	19 (1.9)
T4	85 (6.1)	816 (83.2)
Examined lymph nodes	16 (10,23)	10 (7,14)
Positive lymph nodes		
As continuous	1 (0,3)	1 (0,4)
0	686 (49.5)	366 (37.3)
1-2	306 (22.1)	230 (23.4)
3-6	228 (16.4)	236 (24.1)
7-15	137 (9.9)	126 (12.8)
≥16	30 (2.2)	23 (2.3)
LNR	0.26 (0.00,0.20)	0.17 (0.00,0.45)
Neoadjuvant chemotherapy		NA
Yes	769 (55.4)	
No/Unknown	618 (44.6)	
Neoadjuvant radiotherapy		NA
Yes	586 (42.2)	
No/Unknown	801 (57.8)	
Adjuvant chemotherapy		NA
Yes	981 (70.7)	
No/Unknown	406 (29.3)	
Adjuvant radiotherapy		NA
Yes	778 (56.1)	
No/Unknown	609 (43.9)	
Follow up month*	61 (58-64)	60 (60-60)

NOS, not otherwise specified; NA, not available.

#includes Asian/Pacific Islander, American Indian/Alaska Native.

*median (95% confidence interval).

### ELN and stage migration

**Figure 2:**
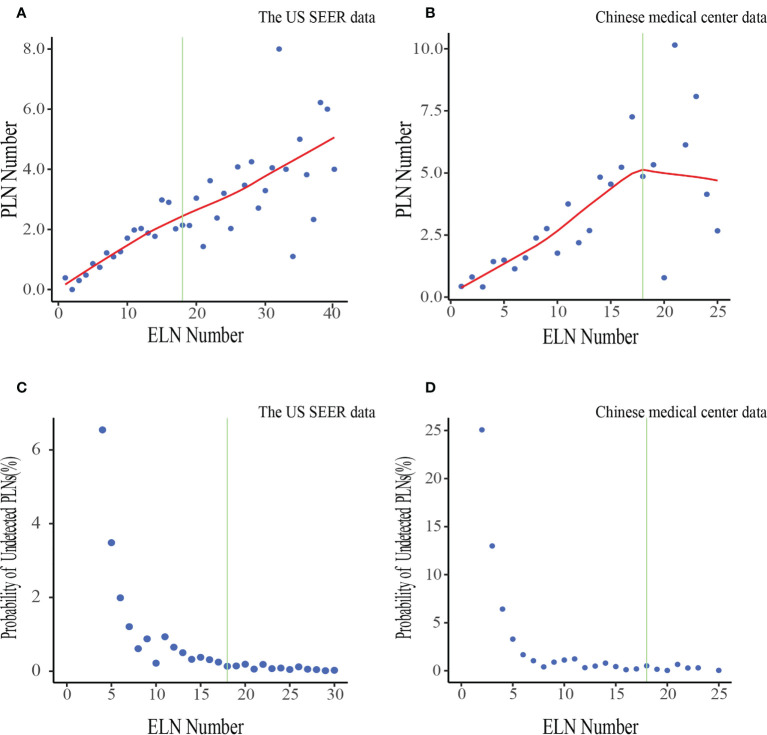
Associations of ELN number with the mean number of PLNs and the probability of undetected PLNs in the US data **(A, C)** and Chinese data **(B, D)**. LOWESS smoother-fitted curves are shown in red. The structural breaks associated with survival in the US data(ELN =18) are shown in green. ELN, Examined lymph node; PLN, Positive lymph node.

The number of PLNs increased while the probability of undetected PLNs decreased as the number of ELNs increased in both two cohorts (**Figure 2**). The odds for negative-to-positive node stage migration increased with more ELNs after multivariable adjustment among both all patients(OR_theUS_1.032,95%CI 1.017–1.046; OR_China_1.034, 95%CI 1.002–1.065)and in most subgroups by gender, age group, ethnicity, differentiation, resection type, tumor size, T stage, Neoadjuvant chemotherapy/radiotherapy administration (**Supplementary Table 1**), Accordingly. The association with stage migration was stronger in T4 patients in the China cohort according to interaction tests.

### ELN and overall survival

A greater number of ELNs was positively associated with better overall survival among both all patients (HR_theUS_0.970,95%CI 0.961-0.979;HR_China_0.960,95%CI 0.940-0.980) and patients in most subgroups, after controlling for other prognostic factors, including gender, age group, ethnicity, differentiation, resection type, tumor size, T stage, positive nodes count, management of adjuvant chemotherapy/radiotherapy administration (**Supplementary Table 2**). Of note, although the association was significant in node-negative Siewert type II AEG declared in the US cohort(HR_theUS_0.974,95%CI 0.960–0.989;HR_China_0.967,95%CI 0.931–1.004), it persisted in both countries in node-positive disease(HR_theUS_0.967,95%CI 0.955–0.979;HR_China_0.953,95%CI 0.930–0.977). The strength of the association was stable in subgroups of the same stratification factors between the two cohorts despite a few statistically significant interaction test results.

### Cut point analysis and validation

**Figure 3:**
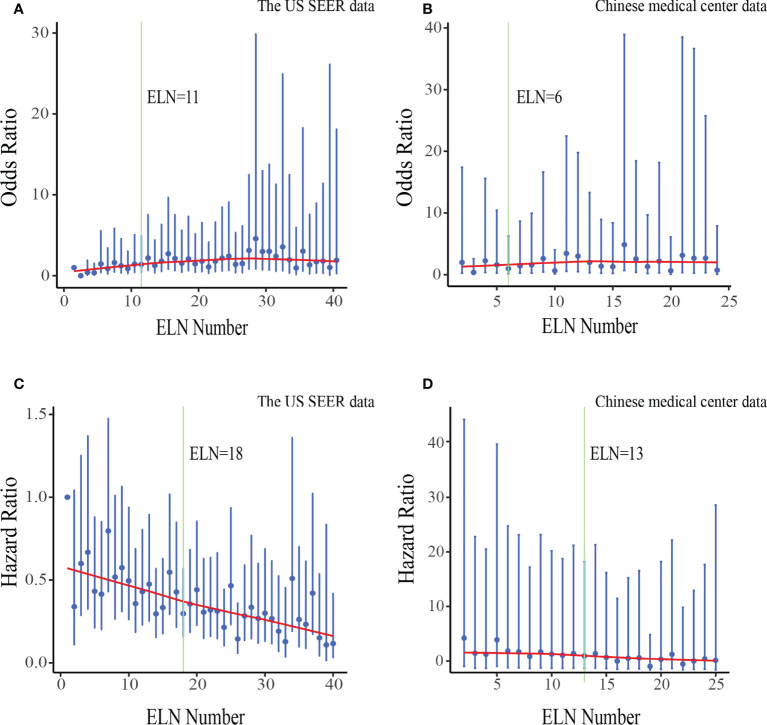
Associations of ELN number with odds ratio(OR) of negative-to-positive node stage migration and hazard ratio(HR) of overall survival in the US data **(A, C)** and Chinese data **(B, D)**. ORs and HRs and the corresponding 95% confidence intervals are shown in blue. LOWESS smoother-fitted curves are shown in red. The structural breaks are shown in green. ELN, Examined lymph node.

The fitting curves and corresponding structural break points for associations of ELN number with OR of negative-to-positive node stage migration and HR of overall survival were displayed in **Figure 3**. We selected 18 ELNs generated from the generalized and representative SEER database as the optimal cut point when all structural break points were basically consistent (varied from 6 to 18).

**Figure 4:**
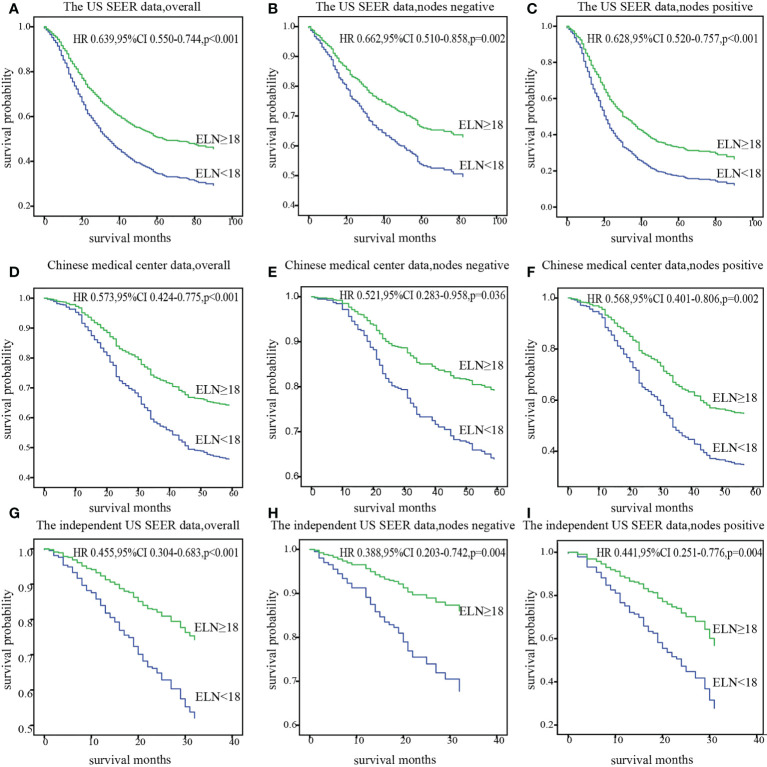
Stratification of overall survival with the cut point of 18 ELNs after adjusting for prognostic factors in patients with overall, nodes negative, and nodes positive diseases in the US data **(A-C)**, Chinese data **(D-F)** and the independent US data **(G-I)**. ELN, Examined lymph node; HR, hazard ratio.

The cut point was first validated in the US SEER data cohort and Chinese medical center data cohort from where it originated, and then in an independent SEER data cohort from 2015 to 2016. Survival analysis confirmed significantly decreased mortality of patients with at least 18 ELNs after adjusting for other prognostic factors in overall, nodes negative and nodes positive.(HR_theUS_0.639,95%CI 0.550–0.744;HR_China_0.573,95%CI 0.424-0.775), consistent result was obtained in the independent SEER cohort (HR 0.455; 95% CI 0.304-0.683) (**Figure 4**).

## Discussion

In the current study, the PLN number at each ELN count was lower in the US than in China. The different ELN and PLN numbers observed might reflect the discrepancy in practice patterns of surgical management and enumeration of lymph nodes varied by surgeons and pathologists between countries. Stage migration analysis showed that more ELNs was associated with a higher proportion of more advanced N stage patients in the overall population of both the US and China cohorts after adjusting for the risk factors associated with lymph node involvement. This association was confirmed by a trend in the mean number of PLNs and the probability of false-negative nodes(undetected PLNs). Examining more lymph nodes reduces the risk of not detecting PLNs, which may lead to more complete elimination of malignancy remnants (ie, a potential source of recurrence) to improve long-term survival. Therefore, both western and eastern cohorts suggested a strong correlation between more numbers of ELN and better overall survival in Siewert Type II-III AEG after multivariable adjustment in both overall and nodes negative as well as nodes positive.

Results from observational investigations, like our research, can only suggest association, but not causation. It cannot be concluded any causal relationship between ELN number and overall survival, but dissecting or examining more nodes inferred in favor of survival. Several additional notes on the relationship between ELN number and overall survival exist. First of all, patients observed to be node-negative with few ELNs might include some patients who actually have node-positive disease. For node-positive patients, patients with a larger number of lymph node samples might include some patients who received appropriate adjuvant therapy for correct staging. Secondly, more ELNs are usually achieved in larger professional institutions within a quality-based setting where survival is generally better.

The key issue in this study is an adequate threshold for ELN numbers. Patients with less than the threshold ELNs might have a higher residual risk of PLNs and poorer survival. It should be emphasized that this is not to encourage more extended lymphadenectomy. Results from earlier randomized study (9) suggested that compared with standard lymphadenectomy, extended lymphadenectomy with largely varying ELNs was not associated with improved overall survival. Although extended lymphadenectomy did not increase postoperative mortality, it tended to increase complications (20, 21). Compared with the thresholds determined by us, Further prospective/randomized studies on the association of excess ELN number with survival might be warranted due to a small number of patients had a relatively large number of ELNs. So far, there is no uniform conclusion yet on the threshold ELN number that could best address both stage migration and long-term survival in AEG, nor has recommendations on ELN number been made in National Comprehensive Cancer Network (NCCN) guidelines, although several retrospective studies in esophageal cancer or gastric cancer have attempted to set a benchmark (22–25). A study on esophageal cancer found that the recommended cut point for lymph node harvesting for esophageal adenocarcinoma was 15, and 14 was determined for esophageal squamous cell carcinoma (26). Notably, another study of gastric adenocarcinoma based on Western and Eastern populations determined a minimal lymph node number of 16 and a potential optimal cut-points of 33 (25). An optimal threshold of 18 ELNs was identified in our study, which was validated in all cohorts. This threshold could be considered as one of the reference metrics for defining lymph node undersampling.

To our knowledge, this study is currently a large study using a real-world dataset with robust statistics on this type of problem. We tried to emphasize two main points. First, ELN numbers were associated with prognosis in Siewert Type II-III AEG, therefore, surgeons and pathologists should spare no effort to explore lymph nodes. We further found that an optimal threshold or range to assess the integrity of AEG lymph node sampling increasingly needs to be established. For example, the NCCN guidelines (3) recommend that patients who have received perioperatively chemoradiation or chemotherapy, postoperative chemotherapy is a category one recommendation for patients with completely resected, node-negative, or node-positive disease. The number of harvested lymph node might be one of the evaluation criteria.

Some limitations also deserve mention in the present study. First, as a retrospective study, despite our study controlled for a number of prognostic factors that may have influenced the outcome, confounding by unknown factors could not be ruled out. Second, there are limitations in the SEER database, such as the lack of information on lymph node stations, therefore we did not investigate the impact of lymph node stations on prognosis in this study. Third, Siewert type II-III AEG was staged using the gastric cancer staging system in the seventh edition of the American Joint Committee on Cancer (AJCC) Cancer Staging Manual in our study, and the effect of the latest TNM on the results requires further validation. Fourth, neither neoadjuvant therapy was routinely performed nor information on adjuvant chemotherapy and radiotherapy was available in China cohort.

## Conclusion

In conclusion, more ELNs are associated with accurate nodal staging(negative-to-positive) as well as higher overall survival in resected Siewert types II-III AEG in this observational study, We recommend 18 ELNs as the optimal cut point for the quality assessment of postoperative lymph node examination or prognostic stratification in clinical practice. Our findings provide an important reference for quality metrics in population-based standardized treatment of Siewert types II-III AEG.

## Data availability statement

The original contributions presented in the study are included in the article/**Supplementary Material**. Further inquiries can be directed to the corresponding authors.

## Ethics statement

The studies involving human participants were reviewed and approved by Ethics Committee of The First Hospital of Anhui University. The patients/participants provided their written informed consent to participate in this study.

## Author contributions

BD and JY collected the clinical information of patients, performed the statistical analysis, and completed the writing of the manuscript. LZ and PL assisted in collecting the patients’ clinical information and review the manuscript. ES helped them. AR reviewed the article. AX, XW and ZW designed the main study and critically revised the manuscript. All authors contributed to the article and approved the submitted version.

## Funding

Anhui Provincial Key Research and Development Project(202104j07020029); Natural Science Foundation of Anhui Province(2108085QH337).

## Acknowledgments

The Surveillance, Epidemiology, and End Results (SEER) database is a federally funded, publicly available cancer reporting system that represents a collaboration between the Centers for Disease Control and Prevention, the National Cancer Institute, and regional and state cancer registries. We greatly appreciate the input of all those associated with it.

## Conflict of interest

The authors declare that the research was conducted in the absence of any commercial or financial relationships that could be construed as a potential conflict of interest.

## Publisher’s note

All claims expressed in this article are solely those of the authors and do not necessarily represent those of their affiliated organizations, or those of the publisher, the editors and the reviewers. Any product that may be evaluated in this article, or claim that may be made by its manufacturer, is not guaranteed or endorsed by the publisher.
