# Supplementary material for: Impact of examined lymph node number on accurate nodal staging and long-term survival of resected Siewert type II-III adenocarcinoma of the esophagogastric junction: A large population-based study
Source: Front Oncol. 2022 Oct 31;12:979338. doi: 10.3389/fonc.2022.979338 (PMC9659734; doi:10.3389/fonc.2022.979338)
Supplement: Supplementary file 1 [file DataSheet_1.docx]

**Supplementary Table 1** Association of examined lymph nodes(≥1,as a continuous variable) with negative-to-positive node stage migration

| Stratification | The US SEER data | | | Chinese medical center data | | |
| --- | --- | --- | --- | --- | --- | --- |
|  | OR(95%CI) | *P*_OR_ | *P** | OR(95%CI) | *P*_OR_ | *P** |
| overall | 1.032(1.017-1.046) | **<0.001** |  | 1.033(1.002-1.065) | **0.034** |  |
| Gender |  |  | 0.634 |  |  | 0.702 |
| Male | 1.035(1.019-1.051) | **<0.001** |  | 1.040(1.004-1.076) | **0.027** |  |
| Female | 1.005(0.971-1.042) | 0.764 |  | 1.016(0.944-1.093) | 0.668 |  |
| Age |  |  | 0.216 |  |  | 0.133 |
| <55 | 1.030(0.999-1.061) | 0.054 |  | 1.170(1.057-1.296) | **0.002** |  |
| 55-65 | 1.043(1.015-1.072) | **0.003** |  | 1.012(0.963-1.063) | 0.648 |  |
| 65-75 | 1.010(0.985-1.036) | 0.432 |  | 1.029(0.978-1.083) | 0.273 |  |
| ≥75 | 1.071(1.026-1.118) | **0.002** |  | 0.999(0.893-1.118) | 0.990 |  |
| Ethnicity |  |  | 0.894 | NA |  |  |
| White | 1.033(1.017-1.049) | **<0.001** |  |  |  |  |
| Black | 1.291(1.029-1.619) | **0.027** |  |  |  |  |
| Others# | 1.022(0.975-1.071) | 0.370 |  |  |  |  |
| differentiation |  |  | 0.835 |  |  | 0.999 |
| Well | 1.071(0.967-1.187) | 0.189 |  | NE |  |  |
| Moderately | 1.024(1.000-1.048) | 0.051 |  | 1.040(0.986-1.097) | 0.154 |  |
| Poorly/Undifferentiated | 1.037(1.017-1.057) | **<0.001** |  | 1.027(0.989-1.066) | 0.170 |  |
| Surgery |  |  | 0.791 |  |  | 0.756 |
| Partial/subtotal/hemi- gastrectomy | 1.037(1.015-1.059) | **0.001** |  | 0.983(0.813-1.189) | 0.861 |  |
| Near-total/total gastrectomy | 1.022(0.992-1.053) | 0.150 |  | 1.032(1.000-1.064) | **0.049** |  |
| Gastrectomy (NOS) | 1.029(1.001-1.058) | **0.041** |  | NA |  |  |
| Tumor size |  |  | 0.877 |  |  | 0.325 |
| <40 | 1.030(1.009-1.052) | **0.006** |  | 1.063(1.003-1.126) | **0.040** |  |
| 40-60 | 1.035(1.007-1.064) | **0.014** |  | 1.028(0.973-1.086) | 0.332 |  |
| >60 | 1.034(1.003-1.066) | **0.033** |  | 1.029(0.978-1.082) | 0.266 |  |
| T stage |  |  | 0.283 |  |  | **0.028** |
| T1 | 1.021(0.981-1.063) | 0.302 |  | 0.966(0.681-1.370) | 0.847 |  |
| T2 | 1.019(0.980-1.059) | 0.344 |  | 1.140(0.979-1.328) | 0.091 |  |
| T3 | 1.036(1.018-1.056) | **<0.001** |  | NE |  |  |
| T4 | 1.112(1.007-1.229) | **0.035** |  | 1.043(1.009-1.078) | **0.013** |  |
| Neoadjuvant chemotherapy |  |  | 0.911 | NA |  |  |
| Yes | 1.030(1.011-1.049) | **0.001** |  |  |  |  |
| No/Unknown | 1.034(1.009-1.059) | **0.007** |  |  |  |  |
| Neoadjuvant radiotherapy |  |  | 0.501 | NA |  |  |
| Yes | 1.030(1.009-1.052) | **0.006** |  |  |  |  |
| No/Unknown | 1.037(1.016-1.058) | **<0.001** |  |  |  |  |

Abbreviations: NOS, not otherwise specified; OR, odds ratio; CI, confidence interval; NA, not available; NE, not estimable

#includes Asian/Pacific Islander, American Indian/Alaska Native

* *P* value for interaction test

**Supplementary Table 2** Association of examined lymph nodes(≥1,as a continuous variable) with overall survival

| Stratification | The US SEER data | | | Chinese medical center data | | |
| --- | --- | --- | --- | --- | --- | --- |
|  | HR(95%CI) | *P*_HR_ | *P** | HR(95%CI) | *P*_HR_ | *P** |
| overall | 0.970(0.961-0.979) | **<0.001** |  | 0.960(0.940-0.980) | **<0.001** |  |
| Gender |  |  | **0.027** |  |  | 0.149 |
| Male | 0.966(0.957-0.977) | **<0.001** |  | 0.966(0.944-0.988) | **0.003** |  |
| Female | 0.985(0.963-1.007) | 0.187 |  | 0.920(0.875-0.967) | **0.001** |  |
| Age |  |  | 0.896 |  |  | **0.037** |
| <55 | 0.953(0.931-0.975) | **<0.001** |  | 0.972(0.920-1.027) | 0.308 |  |
| 55-65 | 0.964(0.946-0.982) | **<0.001** |  | 0.952(0.920-0.985) | **0.004** |  |
| 65-75 | 0.974(0.959-0.990) | **0.001** |  | 0.947(0.914-0.981) | **0.002** |  |
| ≥75 | 0.971(0.948-0.994) | **0.014** |  | 0.961(0.894-1.032) | 0.270 |  |
| Ethnicity |  |  | 0.174 | NA |  |  |
| White | 0.973(0.963-0.983) | **<0.001** |  |  |  |  |
| Black | 0.921(0.851-0.997) | **0.042** |  |  |  |  |
| Others# | 0.965(0.931-1.002) | 0.060 |  |  |  |  |
| differentiation |  |  | 0.424 |  |  | 0.866 |
| Well | 0.973(0.935-1.013) | 0.183 |  | NE |  |  |
| Moderately | 0.976(0.960-0.992) | **0.004** |  | 0.963(0.928-0.999) | **0.047** |  |
| Poorly/Undifferentiated | 0.966(0.954-0.978) | **<0.001** |  | 0.959(0.935-0.983) | **0.001** |  |
| Surgery |  |  | 0.656 |  |  | 0.463 |
| Partial/subtotal/hemi- gastrectomy | 0.968(0.954-0.983) | **<0.001** |  | 0.801(0.617-1.040) | 0.096 |  |
| Near-total/total gastrectomy | 0.960(0.941-0.979) | **<0.001** |  | 0.961(0.941-0.981) | **<0.001** |  |
| Gastrectomy (NOS) | 0.976(0.959-0.993) | **0.007** |  | NA |  |  |
| Tumor size |  |  | 0.772 |  |  | 0.053 |
| <40 | 0.967(0.953-0.981) | **<0.001** |  | 1.001(0.958-1.046) | 0.969 |  |
| 40-60 | 0.976(0.959-0.994) | **0.009** |  | 0.946(0.913-0.981) | **0.003** |  |
| >60 | 0.963(0.944-0.982) | **<0.001** |  | 0.934(0.905-0.963) | **<0.001** |  |
| T stage |  |  | 0.219 |  |  | **0.033** |
| T1 | 0.970(0.942-0.999) | **0.043** |  | 0.923(0.751-1.134) | 0.444 |  |
| T2 | 0.966(0.940-0.992) | **0.012** |  | 0.912(0.794-1.047) | 0.191 |  |
| T3 | 0.973(0.962-0.984) | **<0.001** |  | NE |  |  |
| T4 | 0.933(0.896-0.972) | **0.001** |  | 0.953(0.932-0.973) | **<0.001** |  |
| Nodal status |  |  | 0.335 |  |  | 0.337 |
| N0 | 0.974(0.960-0.989) | **0.001** |  | 0.967(0.931-1.004) | 0.076 |  |
| N_X_& | 0.967(0.955-0.979) | **<0.001** |  | 0.953(0.930-0.977) | **<0.001** |  |
| Adjuvant chemotherapy |  |  | 0.113 | NA |  |  |
| Yes | 0.967(0.957-0.978) | **<0.001** |  |  |  |  |
| No/Unknown | 0.975(0.957-0.994) | **0.010** |  |  |  |  |
| Adjuvant radiotherapy |  |  | 0.157 | NA |  |  |
| Yes | 0.969(0.957-0.981) | **<0.001** |  |  |  |  |
| No/Unknown | 0.974(0.960-0.989) | **0.001** |  |  |  |  |

Abbreviations: NOS, not otherwise specified; HR, hazard ratio; CI, confidence interval; NA, not available; NE, not estimable

#includes Asian/Pacific Islander, American Indian/Alaska Native

& at least one positive lymph node

* *P* value for interaction test
